# Antioxidants and Oxidative Stress in Children: Influence of Puberty and Metabolically Unhealthy Status

**DOI:** 10.3390/antiox9070618

**Published:** 2020-07-15

**Authors:** Azahara I. Rupérez, María D. Mesa, Augusto Anguita-Ruiz, Esther M. González-Gil, Rocío Vázquez-Cobela, Luis A. Moreno, Ángel Gil, Mercedes Gil-Campos, Rosaura Leis, Gloria Bueno, Concepción M. Aguilera

**Affiliations:** 1 GENUD Research Group, Instituto Agroalimentario de Aragón (IA2), Instituto de Investigación Sanitaria (IIS) Aragón, University of Zaragoza, 50013 Zaragoza, Spain; airuperez@unizar.es (A.I.R.); esthergg@ugr.es (E.M.G.-G.); lmoreno@unizar.es (L.A.M.); 2 Department of Biochemistry and Molecular Biology II, Institute of Nutrition and Food Technology “José Mataix”, Center of Biomedical Research, University of Granada, Armilla, 18016 Granada, Spain; mdmesa@ugr.es (M.D.M.); augustoanguita@ugr.es (A.A.-R.); agil@ugr.es (Á.G.); caguiler@ugr.es (C.M.A.); 3 Instituto de Investigación Biosanitaria ibs, 18014 Granada, Spain; 4 CIBEROBN, (Physiopathology of Obesity and Nutrition Network) Institute of Health Carlos III (ISCIII), 28029 Madrid, Spain; cobela.rocio@gmail.com (R.V.-C.); mercedes_gil_campos@yahoo.es (M.G.-C.); mariarosaura.leis@usc.es (R.L.); 5 Pediatric Gastroenterology, Hepatology and Nutrition Unit. Pediatric Department, Clinic University Hospital of Santiago, Instituto de Investigación Sanitaria de Santiago (IDIS), 15706 Santiago de Compostela, Spain; 6 Metabolism and Investigation Unit, Reina Sofia University Hospital, Maimónides Institute of Biomedicine Research of Córdoba (IMIBIC), University of Córdoba, 14004 Córdoba, Spain; 7 Unit of Investigation in Nutrition, Growth and Human Development of Galicia, Pediatric Department, University of Santiago de Compostela, 15782 Santiago de Compostela, Spain; 8 Pediatric Endocrinology Unit, Clinic University Hospital Lozano Blesa, Facultad de Medicina, Universidad de Zaragoza, 50009 Zaragoza, Spain

**Keywords:** carotenoids, tocopherols, oxidized low-density lipoprotein, inflammatory biomarkers, childhood obesity, metabolically healthy, metabolic syndrome

## Abstract

Oxidative stress could help explain the relationship between childhood obesity and a metabolically unhealthy (MU) status. Moreover, puberty could also influence this relationship, since it entails physiological cardiometabolic changes. We aimed to evaluate plasma antioxidants and oxidative stress biomarkers in MU and metabolically healthy (MH) prepubertal and pubertal children and their associations with pro-inflammatory and endothelial damage biomarkers, taking puberty into account. A total of 1444 Spanish children aged 3–17 years (48.9% males, 66% prepubertal, 47.1% with obesity) were recruited. Blood pressure, anthropometric and biochemical parameters were measured, and children were categorized as having a MU or MH status according to risk factors. Retinol, carotenes, tocopherols, total antioxidant capacity (TAC), oxidized low-density lipoprotein and selected pro-inflammatory and endothelial damage biomarkers were analyzed. General linear models adjusted for age, sex, recruitment center and body mass index, partial correlations and stepwise linear regressions were performed. Lower carotenes and tocopherols levels were found in MU than in MH children. Plasma TAC was lower in prepubertal and higher in pubertal children with obesity compared to normal-weight children. Antioxidants and oxidative stress biomarkers showed novel associations with several pro-inflammatory and endothelial damage biomarkers, with pubertal differences, supporting the importance of considering both the antioxidant and oxidative stress status and puberty in the prevention of metabolic diseases in childhood.

## 1. Introduction

Overweight and obesity are increasing worldwide, having become important health issues in childhood [1]. Sedentariness, low physical activity, and unhealthy diets are the greatest contributors to this epidemic [2]. Obesity is frequently associated with the presence of hypertension, dyslipidemia, and insulin resistance, which have been clustered under the term of metabolically unhealthy (MU) status. However, there are also individuals with obesity who do not present any of these cardiometabolic derangements, known as metabolically healthy (MH) [3]. Although there are several available definitions of MU status, there is evidence that both childhood obesity and MU status increase cardiovascular disease risk in adulthood [4,5]. Moreover, systemic inflammation and endothelial damage have also been described in children with obesity [6,7], as well as increased oxidative stress [8,9]. The relationship between oxidative stress, obesity, and cardiometabolic disease seems to be a consequence of low-grade inflammation [10]. In addition, in the presence of excess weight, a decreased antioxidant defense could further contribute to a higher cardiometabolic risk [11]. However, these changes are often complex to detect, due to the homeostatic responses of the organism, as well as to changes associated with childhood development, such as the onset of puberty [12]. Indeed, puberty entails a physiological insulin resistance that influences oxidative stress and inflammation [13,14]. Furthermore, it could accelerate cardiometabolic alterations, since it has been observed that puberty worsens endothelial dysfunction in young patients with type 1 diabetes [15]. Also, puberty reduces insulin sensitivity and triggers hyperinsulinemia, both having a reciprocal relationship with endothelial dysfunction [16]. Taken together, these findings show that puberty influences insulin resistance and oxidative stress, known to contribute to the development and progression of cardiometabolic risk and, particularly, endothelial damage [12].

Children and adolescents with obesity often follow inadequate diets, characterized by a low intake of fruits and vegetables and high intake of sugars and fats, associated with a negative antioxidant status [17,18,19]. Dietary vitamins such as carotenoids and tocopherols are of great interest as bioactive compounds with antioxidant properties and potential positive effects on adipogenesis, lipid, and glucose metabolisms [20,21], since they are involved in lipid metabolism and adipocyte differentiation [22]. Serum circulating concentrations of carotenes have been negatively associated with obesity [23] and metabolic syndrome in children [11], and in adults and adolescents [24]. As for tocopherols, their levels are lower in children with obesity, hypertension [25], and metabolic syndrome [11], and negatively associated with body mass index (BMI), waist circumference (WC) [26], and C reactive protein (CRP) plasma/serum levels [27]. Lower serum or plasma total antioxidant capacity (TAC) has also been associated with metabolic syndrome in children, adolescents and adults [11,28]. In addition, increased circulating oxidized low-density lipoproteins (ox-LDL) plasma levels have already been observed in children with obesity [29]. However, most available studies to date have investigated individual cardiometabolic alterations, type 1 diabetes, or obesity, without considering important aspects such as puberty or studying its influence on their findings. In fact, pubertal children have shown higher plasma levels of lipoperoxidation products than prepubertal children [14]. In addition, puberty has been shown to influence endothelial function and blood antioxidant enzymes in patients with type 1 diabetes [15]. However, broader studies are required to investigate the close relationship between puberty and antioxidant and oxidative stress biomarkers in terms of their impact on MU status risk, understood as the presence of one or more cardiometabolic alterations (hypertension, hyperglycemia, hypertriglyceridemia, low high-density lipoprotein cholesterol (HDL-C) or insulin resistance; as well as its association with inflammation and endothelial damage biomarkers.

The aims of the present study were: (1) To analyze and compare circulating plasma concentrations of retinol, carotenes, tocopherols, TAC, and ox-LDL in prepubertal and pubertal children, (2) to study the association between a MH or MU status and circulating plasma concentrations of the mentioned antioxidants and oxidative stress biomarkers, and (3) to analyze their potential associations with biomarkers of inflammation and endothelial damage, in a group of Spanish children aged 3 to 17 years.

## 2. Materials and Methods

### 2.1. Population

A total of 1444 children (706 males and 738 females), aged 3 to 17 years, were recruited at three Spanish Hospitals (Hospital Universitario Reina Sofía in Córdoba, Hospital Clínico Universitario in Santiago de Compostela, and Hospital Clínico Universitario Lozano Blesa in Zaragoza) in the GENOBOX case-control study [30]. Inclusion criteria were: Caucasian ethnicity, children and adolescents aged 3 to 17 and having normal-weight (controls) or overweight or obesity. Exclusion criteria were: Presence of diabetes mellitus, presence of congenital, chronic, or inflammatory disease, psychomotor disability, use of hormonal medication or other that modifies blood pressure, glucose or lipid metabolism, having performed intense exercise in the 24 h previous to the examination, and/or having participated in a research study in the previous three months. Written informed consent was obtained from parents or caregivers, and children gave their assent, after fully explaining the study protocol. The study protocol was approved by the local Ethics Committees of each participating hospital (Code IDs: Córdoba 01/2017, Santiago 1011/198, Zaragoza 10/2010). The study was performed according to the ethical guidelines of the Edinburgh revision of Declaration of Helsinki (2000).

### 2.2. Clinical and Anthropometric Examination

Clinical features and Tanner stage according to puberty developmental signs were evaluated by clinicians. From the studied population, 953 children were prepubertal (Tanner stage I) and 491 were at higher pubertal stages (Tanner stages II–V) [31].

Weight and height were measured according to standard international procedures with the children barefooted and in their underwear. BMI was calculated as height (m)/weight (kg)^2^. Children where then categorized according to their BMI status as having normal-weight (406 children: 28.1%, of which 53.7% were males), overweight (358 children: 24.8%, of which 41.6% were males) and obesity (680 children: 47.1%, of which 49.9% were males) (Table S1), using the International Obesity Task Force definition [32].

Blood pressure (BP) was measured from the non-dominant upper arm by a trained staff member in a quiet, temperature-controlled room, using an automatic device (Omron M4-I Intellisense, Omron Corporation, Osaka, Japan) after a 20-min rest. Three values were taken at 2-min intervals and the average of the two closest measures was used for further analyses.

### 2.3. Blood Sampling

Blood samples were taken in a fasting state between 8:00 and 11:00 am and were centrifuged immediately. Serum glucose, triacylglycerols (TAG), HDL-C and LDL cholesterol (LDL-C) concentrations were measured in fresh samples using the clinical analysis system Roche Hitachi Modular DPP (Roche, Basel, Switzerland). Fasting serum insulin was determined in fresh samples using an Elecsys Modular E-170 (Roche, Basel, Switzerland). The Homeostasis Model Assessment of Insulin Resistance (HOMA-IR) was calculated based on the published equation: HOMA-IR = fasting glucose (mmol) × fasting insulin (μU/mL)/22.5 [33]. After centrifugation, plasma aliquots and blood erythrocytes were separated and frozen at −80 °C until further analyses.

### 2.4. Plasma Antioxidants and Ox-LDL Analysis

Measurement of plasma concentrations of retinol, carotenes and tocopherols were determined after extraction with 1-propanol by ultra-high-pressure liquid chromatography coupled to mass spectrometry (UHPLC-MS), using methanol 0.1% and isocratic formic as solvent, with a flow of 0.5 mL/min in a ACQUITY UPLCr BEH C18 50 mm column (internal diameter 2.1 mm and particle size 1.7 μm) [34]. Concentrations were expressed in μg/dL for retinol and total carotenes, and in mg/dL for total tocopherols. In addition, plasma carotenes and tocopherols ratio to TAG were also calculated. Plasma TAC was determined using a commercial antioxidant assay kit (Cat no. 709001, Cayman Chemical, Ann Arbor, MI, USA). Plasma ox-LDL (coefficient of variation [CV]: 7.8%) was determined with an enzymatic immunoassay (Cat no. BI-20042, Biomedica Medizinprodukte GmbH & Co KG, Vienna, Austria).

### 2.5. Plasma Pro-Inflammatory and Endothelial Damage Biomarkers Analysis

Plasma pro-inflammatory and endothelial damage biomarkers were measured using commercial LINCOplex TM kits of human monoclonal antibodies (Linco Research, St. Charles, MO, USA) on a Luminex^®^ 200TM System (Luminex Corporation, Austin, TX, USA). Plasma active plasminogen activator inhibitor-1 (aPAI-1) was measured with the HADK1-61K-A kit (CV: 6.6%). Interleukin (IL)-6 (CV: 7.8%), IL-8 (CV: 7.9%), monocyte chemoattractant protein 1 (MCP-1) (CV: 6%), hepatocyte growth factor (HGF) (CV: 3%) and tumor necrosis factor alpha (TNFα) (CV: 7.8%) were measured using the HADK2-61K-B kit. Myeloperoxidase (MPO) (CV: 12.3%), matrix metalloproteinase 9 (MMP-9) (CV: 6.8%) and total PAI-1 (tPAI-1) (CV: 11.8%) were measured with the HCVD1-67 AK kit. High sensitivity CRP (hsCRP) (CV: 4%) was determined by particle-enhanced turbidimetric immunoassay (Dade Behring Inc., Deerfield, IL, USA).

### 2.6. Metabolic Health Definition

Children were classified in relation to the presence of a MU status according to the five published criteria [7,35]: Systolic or diastolic BP ≥90th percentile for age, sex and height; glucose ≥100 mg/dL; TAG >90th percentile for age, sex and race; HDL-C serum concentration <10th percentile for age and sex; and elevated HOMA-IR, as described previously [7]. HOMA-IR cutoffs were ≥2.5 for prepubertal children and ≥3.38 or ≥3.905 for pubertal males or females, respectively, these cutoffs were previously obtained from the present population as described in [36] due to the absence of reference values [37]. A total of 767 children (47.5% males) fulfilled one or more criteria and were thus considered to have a MU status (Tables S1 and S2).

### 2.7. Dietary Assessment

Food frequency consumption was evaluated with a qualitative food frequency questionnaire that included common foods consumed in Spain. The children and their caregivers were interviewed, and consumption frequency of each food item was recorded as never or hardly ever; once, 2–3, 4–6, or >6 times per day; once, 2–4 or 5–6 times per week; and 1–3 times per month. Coded answers were then converted into times per week and standardized Z-scores were obtained to use in further analyses. Groups of individual foods high in carotenes (cooked and raw vegetables, fruit with and without sugar and fruit juices) or tocopherols (nuts, seeds, vegetable oils and mayonnaise) were created by summing their standardized values into new variables, according to the Spanish Food Composition Database [38].

### 2.8. Statistical Analysis

Descriptive statistics, included evaluation of the distribution of continuous variables, were evaluated using the Kolmogorov–Smirnov test and through visualization of histogram plots. Non-normally distributed variables (aPAI-1, carotenes, carotenes/TAG, HGF, hsCRP, IL-6, IL-8, MCP-1, MMP-9, TNFα, and tocopherols/TAG, tPAI-1, ox-LDL) were ln-transformed for statistical analyses. Student’s *t*-test was applied to evaluate differences in the descriptive characteristics of the participants. General linear regression models adjusted by sex, age, recruitment center and BMI were used to compare concentrations of antioxidant biomarkers, ox-LDL and standardized consumption frequency of carotene and tocopherol-rich foods between the study groups (male vs. female, prepubertal vs. pubertal, MH vs. MU, etc.). Partial correlations adjusted by age, sex, and BMI were used to analyze the relationship between antioxidant biomarkers and ox-LDL among themselves and the rest of studied biomarkers (cardiometabolic variables and biomarkers of inflammation and endothelial damage). Finally, forward stepwise multiple regression analysis (including age, sex, recruitment center, and BMI as confounders) were carried out to evaluate what antioxidant and oxidative stress biomarkers (independent variables) could be considered as predictors of inflammatory risk and endothelial damage (dependent variables).

## 3. Results

### 3.1. Antioxidant and Oxidative Stress Biomarkers in Prepubertal and Pubertal Children According to Metabolic Health and Obesity

A description of the participants can be found in Appendix A and Supplementary Table S1. Our analyses showed significantly higher TAC in pubertal compared to prepubertal children independently of BMI (Table 1). Due to these observed differences between prepubertal and pubertal children, as well as to the different cutoffs of the applied criteria to classify the children as MH or MU, all subsequent analyses were stratified by pubertal status. Table 1 also shows the results of the BMI-adjusted general linear models run to study the association between the different antioxidant and oxidative stress biomarkers with MU status. We found significantly lower plasma tocopherols/TAG and carotenes/TAG levels and higher ox-LDL concentrations in prepubertal MU compared with MH children. Pubertal children with a MU status also showed lower concentrations of retinol, tocopherols/TAG and carotenes/TAG concentrations than MH participants.

We also evaluated the association between the assessed vitamins and oxidative stress markers with obesity in order to corroborate previous findings. Differences in plasma biomarker concentrations between children with normal-weight or overweight/obesity are shown in Table S3. We observed higher plasma retinol concentrations in prepubertal children with overweight/obesity compared to normal-weight children, while plasma carotenes/TAG and tocopherols/TAG were significantly lower in both prepubertal and pubertal children with overweight/obesity compared to normal-weight children. TAC showed an opposite behavior in prepubertal and pubertal children, being significantly lower in prepubertal and higher in pubertal overweight/obese children compared with their normal-weight counterparts. No significant differences were observed for ox-LDL between children with normal-weight or overweight/obesity.

**Figure 1:**
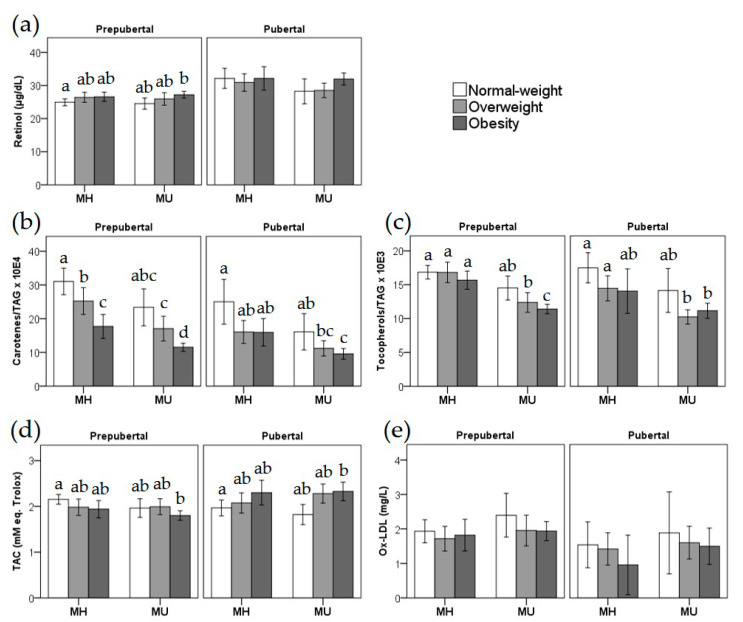
Mean values of plasma retinol (**a**), carotenes/TAG (**b**), tocopherols/TAG (**c**), TAC (**d**), and ox-LDL (**e**) of prepubertal and pubertal children, according to their weight categories (normal-weight, overweight, and obesity) and metabolic health (healthy or unhealthy) status. Within each pubertal category, different letters indicate significant differences at *p* < 0.05 in pairwise comparisons of the general linear model adjusted for sex, age and recruitment center, (sharing the same letters indicates no significant differences between groups, absence of letters indicates no significant differences between any of the groups). MH: metabolically healthy; MU: metabolically unhealthy; ox-LDL: oxidized low-density lipoprotein; TAC: total antioxidant capacity; TAG: triacylglycerols.

Next, Figure 1 shows the plasma antioxidant vitamins and ox-LDL stratified according to MH/MU status, weight categories (normal-weight, overweight, and obesity), and pubertal status. Plasma retinol values (Figure 1a) vary within a narrow range and show significant differences between prepubertal children with MH normal-weight compared to children with MU obesity. Regarding carotenes/TAG (Figure 1b) and tocopherols/TAG (Figure 1c) ratios, the figure illustrates how obesity and MU status display a cumulative effect in these antioxidant markers, which are at significantly lower values in children with overweight or obesity and a MU status, both in prepubertal and pubertal children. In addition, it can be observed how TAC (Figure 1d) behaves in an opposite manner in prepubertal vs. pubertal children, which depends on weight rather than MU status. Finally, ox-LDL does not show statistically significant differences among the compared groups (Figure 1e).

### 3.2. Evaluation of the Dietary Sources of Carotenes and Tocopherols and Their Influence on Carotene/TAG and Tocopherol/TAG Levels

Next, we compared the standardized consumption frequency of carotene- and tocopherol-rich foods between children with or without excess weight and MU status (Table 2). We observed a significantly lower consumption frequency of tocopherol-rich foods in MU compared with MH prepubertal children, independently of BMI and no differences in the consumption frequency of carotene-rich foods. The partial correlation analysis (adjusted by age, sex and BMI) between the consumption frequency of carotene or tocopherol-rich foods and plasma carotene/TAG and tocopherols/TAG concentrations, respectively, stratified by the children’s weight status (normal-weight, overweight, obesity) showed a positive association between the standardized consumption frequency of carotene-rich foods and plasma carotene/TAG concentrations (R = 0.367, *p* = 0.009) exclusively in normal-weight children, which was only confirmed in the pubertal group (R = 0.727, *p* = 0.005) in the puberty-stratified analysis (data not shown).

### 3.3. Exploring the Relationship between Antioxidants and Oxidative Stress Biomarkers and Cardiometabolic, Pro-inflammatory and Endothelial Damage Markers

A partial correlation analysis was conducted to evaluate the relationship between antioxidant and oxidative stress biomarkers among themselves (Table 3). As could be expected, carotenes/TAG and tocopherols/TAG were strongly and positively correlated with each other (R > 0.500, *p* < 0.001) in the total and puberty stratified analyses. Less strong correlations were observed between retinol and tocopherols/TAG (R > 0.200, *p* < 0.001). Retinol and tocopherols/TAG were also negatively correlated with ox-LDL when analyzing the total sample (*p* < 0.001).

When studying the correlation between the analyzed antioxidants and oxidative stress biomarkers and cardiometabolic variables (Table 4), we found a negative correlation between carotenes/TAG and tocopherols/TAG and insulin and HOMA-IR (all R = −0.170, *p* < 0.01) in the total sample and puberty-stratified analyses. Retinol was positively correlated with cholesterol and TAG in all analyses (R > 0.140, *p* < 0.001). TAC was negatively correlated with SBP in the total sample (R = −0.087, *p* < 0.01) and prepubertal children (R = −0.103, *p* < 0.01), negatively correlated with HDL-C in the total sample (R = −0.86, *p* < 0.01) and pubertal children (R = −0.118, *p* < 0.05), and positively correlated with TAG in the total sample (R = 0.087, *p* < 0.01) and pubertal children (R = 0.162, *p* < 0.01). (Table 4).

Finally, we examined the association between the analyzed antioxidants and oxidative stress biomarkers and pro-inflammatory and endothelial damage biomarkers, in the total sample and stratifying by pubertal stage. We first performed a partial correlation analysis to identify potential associations between the studied biomarkers (Table S4). In view of the multiple observed associations, we conducted a forward stepwise multiple regression analysis which results are shown in Table 5. We found a strong positive association between ox-LDL and tPAI-1 in both the total and puberty stratified analyses. Retinol was positively associated with MCP-1, TNFα, aPAI-1, and tPAI-1, and negatively associated with hsCRP and MPO in prepubertal children, and in the total sample (with slightly less significant associations). Similarly, carotenes/TAG values were negatively associated with aPAI-1 and tPAI-1. Plasma tocopherols/TAG were positively associated with TNFα and plasma TAC was negatively associated with MPO in prepubertal children and in the total sample. Concerning significant associations in pubertal children, a strong positive association was found between TAC and MCP-1 and TNFα, paralleled by a less significant positive association between ox-LDL and MCP-1. Also, negative associations were observed between plasma tocopherols/TAG and IL-8 concentrations, and between TAC and tPAI-1 in both pubertal children and the total sample.

## 4. Discussion

In the present study we aimed to evaluate differences in antioxidant and oxidative stress biomarkers in MU compared with MH children according pubertal status, as well as their association with pro-inflammatory and endothelial damage biomarkers. Our findings show that pubertal children had higher plasma TAC values than prepubertal children, independently of BMI. We also observed lower carotenes/TAG and tocopherols/TAG plasma values in prepubertal and pubertal MU children compared to MH children, while ox-LDL concentrations were higher only in prepubertal MU children compared to their healthy counterparts, independently of BMI. In addition, several significant associations were found between the antioxidants and oxidative stress biomarkers and several biomarkers of inflammation and endothelial damage.

### 4.1. Pubertal Differences in Antioxidant and Oxidative Stress Biomarkers in Children with Obesity or MU Status

Our results support the existence of pubertal differences in the levels of selected biomarkers of antioxidant and oxidative stress status in response against excess weight and MU status. According to our findings, the prepubertal period appears as a more sensitive stage, in which plasma antioxidants and TAC levels were negatively correlated with pro-inflammatory and endothelial damage biomarkers. Thus, prepubertal children seem to be in a situation in which endothelial damage is taking place in parallel to the lower antioxidant levels and higher ox-LDL, while responses against oxidative stress that would increase TAC are not yet so evident. In this scenario, mechanisms contributing to endothelial dysfunction include glucotoxicity, lipotoxicity, and inflammation [39]. Indeed, elevated free fatty acid levels, present in individuals with diabetes, obesity or dyslipidemias may lead to lipotoxicity [40,41]. Inflammation is thought to act as a catalyst in cardiovascular disease, being able to enhance endothelial damage [12].

In contrast, the pubertal period shows a different pattern. Indeed, most correlations found in prepubertal children are changed in pubertal children, and different ones arise. These are mainly observed for TAC and ox-LDL and mostly related to inflammation. It should be reminded that changes occurring during puberty are closely related to oxidative stress and inflammation [42], which could induce antioxidant defenses. A previous study performed in adolescents showed puberty to be related to oxidative stress [14]. However, other studies have not found differences in antioxidant response mechanisms between healthy children and adolescents [43,44]. Nevertheless, homeostatic responses make difficult to conclude whether these children trigger or not a sufficient response against the occurring alterations [45]. As already mentioned, puberty reduces insulin sensitivity and enhances hyperinsulinemia, both leading to endothelial dysfunction [16]. It seems that the proinflammatory state is more evident in pubertal children. However, larger longitudinal studies are needed to confirm our preliminary findings and propose potential mechanisms, since there are few studies to date, with a limited sample size.

### 4.2. Plasma Retinol Levels Are Decreased in Pubertal Children with MU Status and Correlated with Pro-Inflammatory and Endothelial Damage Biomarkers

In accordance with a previous study [46], we have observed higher retinol plasma concentrations in prepubertal children with obesity. A positive association between vitamin A and BMI, BMI-for-age, waist–height ratio (WHR), and abdominal fat was found in Mexican school-aged children [27]. However, other authors observed lower serum retinol concentrations in children with obesity [47,48], also associated negatively with BMI, WC, and glucose, and positively with HDL-C concentrations [48]. Moreover, we found significantly lower retinol plasma concentrations in pubertal MU children when compared with their healthy counterparts. In contrast with previous studies that found no differences or positive associations between retinol and metabolic syndrome (not MU status) in children and adolescents, as reviewed by Beydoun et al., [24]. Similarly, a previous study also reported the association between retinol deficiency and hepatic steatosis in children [49]. Hence, the role of retinol in cardiometabolic alterations remains unclear.

Concerning the potential role of retinol in cardiovascular risk, our study reveals interesting findings. We observe significant associations between plasma retinol and several pro-inflammatory and endothelial damage biomarkers, but mainly in prepubertal children. We found a negative correlation between retinol and hsCRP and MPO in the prepubertal and total sample. Of these, the finding regarding hsCRP is in accordance with previous studies in adults [50] and children [51], and the correlation with MPO could be the first to be reported. However, we also observed positive associations between retinol and MCP-1, aPAI-1, and tPAI-1 in the prepubertal and total samples that cannot be explained.

### 4.3. Plasma Carotenes/TAG Levels Are Decreased in Children with Obesity or MU Status and Negatively Correlated with Pro-inflammatory and Endothelial Damage Biomarkers, Insulin and HOMA-IR

Our results show a cumulative effect of obesity and MU status in the lowering of carotene/TAG levels (Figure 1B). Indeed, carotene plasma levels have been previously found to be negatively associated with obesity in children [11,23] and to anthropometric parameters (BMI, WC, FM) [47]. Specifically, plasma β-carotene levels were also inversely correlated with body weight but not to plasma total lipids in children with obesity [52]. Available literature supports excess fat could be acting as a trap for β-carotene, decreasing its circulating concentrations in obesity [53], which is in line with the present data and may explain the lower plasma TAC observed in prepubertal children. Indeed, we found a positive correlation between consumption frequency of carotene-rich foods and plasma levels of carotenes/TAG, exclusively in normal-weight children. A previous study found no differences in reported intake of β-carotene, α-tocopherol, fruit, or vegetables between children with or without obesity [54], while other authors have described a significant correlation between fruit and vegetable intake (carotenoids in diet) and plasma carotenoid concentrations in children only after adjusting for BMI [55]. Compared with reference values [56], we found a high prevalence of carotene deficiency in our studied population.

A potential interpretation of our findings is that the higher cardiometabolic risk could be a consequence of the lower plasma carotenes availability, which could increase oxidation of circulating lipids promoting endothelial damage. Accordingly, we observed for the first time a negative association between carotenes/TAG and endothelial damage biomarkers aPAI-1 and tPAI-1. Plasma carotenes/TAG were also negatively correlated with insulin and HOMA-IR, which agrees with a previous study that showed serum β-carotene levels to be negatively correlated with glycaemia in adolescents with obesity and MU status [57].

### 4.4. Plasma Tocopherols/TAG Levels Are Decreased in Children with Obesity or MU Status and Negatively Correlated with Pro-inflammatory and Endothelial Damage Biomarkers, Insulin, and HOMA-IR

We also found lower tocopherol-rich foods consumption in prepubertal MU children compared with MH children, independently of BMI, with no differences between children with or without obesity, as previously reported [54]. A previous study has found plasma α-tocopherol to be negatively associated with BMI and WC in children and adolescents with obesity [26]. However, beyond obesity, our findings show an additional effect of MU status in the lower tocopherols/TAG levels, according to previous data in children with metabolic syndrome [11]. In the present study, we found a negative correlation of plasma tocopherols/TAG levels and insulin and HOMA-IR values. Similarly, vitamin E corrected for plasma lipids was previously reported to be negatively associated with BMI, WHR, abdominal fat, insulin and CRP (*p* < 0.05) in school-aged children [27]. However, we did not observe any correlation with SBP, as previously reported in hypertensive adolescents [25]. Finally, tocopherols/TAG levels were also negatively associated with IL-8 in the pubertal and total samples, and negatively correlated with ox-LDL in all analyses. In contrast, tocopherols/TAG levels were positively associated with TNFα in prepubertal children and the total sample. To the best of our knowledge, this is the first time these associations are observed in children.

### 4.5. Plasma TAC Levels Are Lower in Prepubertal and Higher in Pubertal Children with Obesity, Not Influenced by MU Status and Positively Correlated with MCP-1 Plasma Concentration

In the present study, prepubertal and pubertal children show different patterns of results. We found lower plasma TAC in prepubertal children with excess weight, which is in line with previous studies reporting lower TAC in children with obesity aged 6–16 y [9] and 2–11 y [58]. But, in contrast, we found higher TAC in pubertal children with overweight/obesity than in normal-weight participants. Similarly, a previous study found higher serum total antioxidant status (TAS) in obese children aged 7–17, which was not significantly correlated with BMI [8]. However, the observed differences for TAC were absent when comparing MH and MU children in the BMI-adjusted analysis, maybe indicating the influence of fat mass. According to our knowledge, this is the first study comparing plasma antioxidant state between MH and MU children taking puberty into account. Therefore, more studies are needed to confirm the present data.

Regarding the association analyses, our results show plasma TAC to be positively associated with inflammatory biomarkers MCP-1 and TNFα, and negatively associated with the endothelial damage biomarkers tPAI-1 (in the pubertal and total samples) and MPO (in the prepubertal and total samples). This could be an additional hint on the differential responses observed in prepubertal and pubertal children.

### 4.6. Plasma ox-LDL Levels Are Increased in Prepubertal MU Children and Positively Correlated with PAI-1

In the present study we have observed increased ox-LDL concentrations in prepubertal children with MU status compared with their healthy counterparts. In line with this, increased levels of ox-LDL, which play a key role in the development of atherosclerosis from early ages, have been consistently found in children with obesity [29,59,60]. Also, ox-LDL have been associated with cardiometabolic risk factors [61] and decreased after a 10-week weight loss program in children with obesity [62]. Consistently with these findings and for the first time, to our knowledge, we observed a strong positive association between ox-LDL and tPAI-1 in all analyses. Indeed, previous studies have observed how ox-LDL could activate PAI-1 transcription [63]. In addition, ox-LDL was also negatively correlated with HDL-C, and positively correlated with plasma glucose in our population, further supporting the role of this oxidative stress biomarker as an indicator of an unhealthy cardiometabolic status.

### 4.7. Prevention Strategies

Dietary total antioxidant capacity has been negatively associated to BMI and total body fat only in children and adolescents with obesity [28]. In view of the present findings, the two most straightforward strategies to overcome the increased risk of MU status are weight loss and antioxidant supplementation [19]. Indeed, a weight loss intervention in adolescents was able to increase lipid-corrected β-carotene and α-tocopherol plasma concentrations [52,64] and lead to reduced ox-LDL levels in children with obesity [62]. Moreover, a physical activity program was also able to lower endothelial dysfunction and oxidative stress markers in adolescents with metabolic syndrome [65]. However, whereas in animal models, β-carotene supplementation has been shown to reduce obesity, by leading to downregulation of adipogenic genes [66], randomized control trials have not proven the usefulness of β-carotene supplementation in the prevention of metabolic syndrome. Nevertheless, children with obesity lost weight after being supplemented with a carotenoid mixture [67], while lycopene, a non-provitamin-A carotenoid, has been shown to reduce BP and oxidative stress biomarkers in adults [68].

While antioxidant supplementation may lead to metabolic benefits [20,69,70,71], perhaps the use of food items or dietary patterns rich in antioxidants would be a more appropriate approach to increase circulating availability of carotenes or tocopherols [72,73]. However, there is not a clear dose–response effect, which suggests individual variability in biomarkers reflecting fruit and vegetable intake [73]. These facts support the need of future studies to investigate whether dietary patterns, i.e., Mediterranean diet, and not individual nutrients, could help prevent MU status and its causing mechanisms.

### 4.8. Limitations and Strenghts

The present work has several limitations. First, since food consumption data were derived from a qualitative food frequency questionnaire, the carotene and tocopherol content of the diet could not be quantitatively determined. Also, the observational cross-sectional design does not allow to establish causality of the findings. In addition, the use of fat mass of the participants instead of BMI could have been of interest in order to adjust the analyses by this variable and should be considered in future studies. Finally, other antioxidant molecules that could influence the findings regarding tocopherols, such as Coenzyme Q10 and vitamin C, were not measured.

As for strengths, this is one of the first studies to analyze the effect of puberty on antioxidant and oxidative stress biomarkers in a large sample of prepubertal and pubertal children. Also, we used standardized data from geographically distant and different centers. Moreover, a large set of biomarkers were analyzed, and children were carefully and individually examined by clinicians to determine their Tanner stage. It is also important that we could include data regarding dietary habits.

## 5. Conclusions

In the present study we confirm the previously reported negative association between carotenes/TAG and tocopherols/TAG and obesity and find, for the first time, a strong negative association between MU status and carotenes/TAG and tocopherols/TAG, independently of BMI and pubertal status. These results support the potential role of these antioxidants in obesity and its complications. Moreover, differences due to pubertal status have been identified, highlighting the lower antioxidant status, related with a worse profile of endothelial damage biomarkers, in prepubertal children, whereas in pubertal children inflammation seems to be predominant.

The current data point to both the prepubertal and pubertal periods as important life stages for oxidative and endothelial damage prevention and treatment, including the promotion of a healthy and physically active lifestyle with a diet high in vitamin-rich foods, in order to avoid the onset and progression of cardiometabolic derangement.

These findings support the need of considering puberty in future studies investigating the impact of oxidative stress and antioxidant status in metabolic diseases in childhood, such as obesity or insulin resistance.

## Figures and Tables

**Table 1:** Plasma antioxidant and oxidative stress biomarkers according to metabolic health and pubertal status.

	All			MH			MU			*p*
	*n*	Mean	SD	*n*	Mean	SD	*n*	Mean	SD	
**Prepubertal**										
Retinol (μg/dL)	705	26.2	7.0	319	25.7	6.6	342	26.6	7.5	0.456
Carotene/TAG 10^4^	705	20.31	19.77	319	25.97	21.94	342	14.07	12.61	0.000
Tocopherols/TAG 10^3^	706	14.3	6.4	320	16.5	6.4	342	12.0	5.6	0.000
TAC (mM eq. Trolox)	781	1.95 **	0.82	359	2.06	0.80	375	1.86 **	0.81	0.336
Ox-LDL (mg/L)	579	1.95	1.88	288	1.86	1.90	259	2.01	1.81	0.002
**Pubertal**										
Retinol (μg/dL)	279	30.9	8.6	102	31.6	8.6	168	30.3	8.6	0.048
Carotene/TAG 10^4^	272	14.49	13.03	102	19.03	14.58	163	10.90	8.54	0.000
Tocopherols/TAG 10^3^	277	12.9	6.1	102	15.4	6.6	168	11.1	5.1	0.000
TAC (mM eq. Trolox)	391	2.16 **	0.90	169	2.07	0.80	209	2.25 **	0.97	0.430
Ox-LDL (mg/L)	189	1.51	1.62	80	1.37	1.53	104	1.60	1.71	0.414

Mean and standard deviation (SD) of the studied biomarkers for children with MH or MU status, stratified by pubertal status. *p*: Statistical significance of the general linear model (adjusted for sex, age, recruitment center and BMI) for MU status (independent variable) and individual biomarkers (dependent variables). ** Statistical significance (*p* < 0.01) of the general linear model (adjusted for sex, age, recruitment center, and BMI) between prepubertal and pubertal children within each category (columns). Carotene/TAG, Tocopherols/TAG and ox-LDL were ln-transformed for statistical analyses. BMI: body mass index; MH: metabolically healthy; MU: metabolically unhealthy; ox-LDL: oxidized low-density lipoprotein; TAC: total antioxidant capacity; TAG: triacylglycerols.

**Table 2:** Standardized consumption frequency of carotene-rich and tocopherol-rich foods in metabolically unhealthy and healthy children, by pubertal status.

	Prepubertal							Pubertal						
	MH			MU				MH			MU			
	*n*	Mean	SD	*n*	Mean	SD	*p*	*n*	Mean	SD	*n*	Mean	SD	*p*
**Carotene-rich foods Z-score**	122	−0.02	2.79	184	−0.16	2.47	0.738	122	−0.51	2.08	162	0.11	2.25	0.152
Cooked vegetables Z-score	122	0.08	1.10	184	−0.14	0.92	0.238	122	−0.17	0.74	162	0.15	1.06	0.024
Raw vegetables Z-score	122	−0.17	1.07	184	−0.11	0.91	0.968	122	−0.15	0.93	162	0.11	0.93	0.412
Fruit (no sugar) Z-score	122	−0.06	1.02	184	0.02	0.97	0.785	122	−0.14	0.93	162	0.10	1.09	0.220
Fruit (sugar) Z-score	122	0.01	0.93	184	0.06	1.10	0.811	122	−0.16	0.66	162	−0.07	0.83	0.464
Juices Z-score	122	0.11	1.02	184	0.02	1.00	0.790	122	0.12	1.08	162	−0.18	0.91	0.115
**Tocopherol-rich foods Z-score**	122	0.34	2.11	184	−0.18	1.91	0.018	122	0.04	1.84	162	0.14	1.77	0.354
Nuts + Seeds Z-score	122	0.14	1.06	184	−0.18	0.88	0.029	122	−0.05	1.00	162	0.13	1.04	0.120
Vegetable oils Z-score	122	0.10	1.00	184	−0.03	0.98	0.374	122	0.11	1.03	162	0.07	0.93	0.946
Mayonnaise Z-score	122	0.10	1.11	184	0.04	1.09	0.144	122	−0.03	0.89	162	−0.05	0.91	0.898

Mean and standard deviation (SD) of the standardized Z-score of the consumption frequency of foods rich in carotenes and tocopherols for children with MH or MU status, stratified by pubertal status. *p* value indicates statistical significance of the general linear model adjusted by sex, age, recruitment center and BMI. MH: metabolically healthy; MU: metabolically unhealthy.

**Table 3:** Partial correlation analyses between analyzed plasma antioxidants and ox-LDL.

	Retinol (μg/dL)		Carotenes/TAG		Tocopherols/TAG		TAC (mM eq.Trolox)	
	*n*	Corr.	*n*	Corr.	*n*	Corr.	*n*	Corr.
**Prepubertal children**								
Retinol (μg/dL)								
Carotenes/TAG	700	0.063						
Tocopherols/TAG	700	0.200 ***	700	0.532 ***				
TAC (mM eq.Trolox)	574	−0.006	574	−0.011	574	0.010		
ox-LDL (mg/L)	572	−0.141 ***	572	−0.041	572	−0.130 **	575	−0.003
**Pubertal children**								
Retinol (μg/dL)								
Carotenes/TAG	266	0.120						
Tocopherols/TAG	272	0.316 ***	266	0.598 ***				
TAC (mM eq.Trolox)	182	−0.011	181	−0.011	182	−0.026		
ox-LDL (mg/L)	181	−0.140	180	−0.261 ***	181	−0.187 *	184	−0.132
**Total sample**								
Retinol (μg/dL)								
Carotenes/TAG	971	0.075 *						
Tocopherols/TAG	977	0.242 ***	971	0.548 ***				
TAC (mM eq.Trolox)	761	−0.026	760	0.028	761	0.022		
ox-LDL (mg/L)	758	−0.125 ***	757	−0.085 *	758	−0.131 ***	764	−0.012

Partial correlation adjusted for sex, age and body mass index in the total samples and separately by pubertal stage, superscript letters indicate statistical significance (* *p* < 0.05, ** *p* < 0.01 y *** *p* < 0.001). All variables were logarithmically transformed for analyses except retinol and TAC. ox-LDL: oxidized low-density lipoprotein; TAC: total antioxidant capacity; TAG: triacylglycerols.

**Table 4:** Partial correlation analyses between plasma antioxidants and ox-LDL and metabolically unhealthy status components.

	SBP (mm Hg)		DBP (mm Hg)		Glucose (mg/dL)		Insulin (mU/L)		HOMA-IR		Chol (mg/dL)		TAG (mg/dL)		HDL-C (mg/dL)	
	*n*	Corr.	*n*	Corr.	*n*	Corr.	*n*	Corr.	*n*	Corr.	*n*	Corr.	*n*	Corr.	*n*	Corr.
**Prepubertal children**																
Retinol (μg/dL)	665	0.014	665	−0.051	700	0.063	673	0.075	673	0.071	700	0.206 ***	700	0.154 ***	690	−0.038
Carotenes/TAG *	665	−0.004	665	−0.021	700	−0.046	673	−0.223 ***	673	−0.210 ***	700	0.050	700	NA	690	0.288 ***
Tocopherols/TAG *	665	−0.052	665	−0.062	700	−0.095 *	673	−0.223 ***	673	−0.213 ***	700	0.030	700	NA	690	0.367 ***
TAC (mM eq.Trolox)	735	−0.103 **	735	−0.031	775	0.046	740	−0.003	739	0.006	776	0.038	776	0.055	774	−0.057
ox-LDL (mg/L) *	544	0.056	544	0.074	577	0.051	555	−0.014	555	−0.013	577	0.022	577	0.062	575	−0.110 **
**Pubertal children**																
Retinol (μg/dL)	266	0.023	266	0.099	273	0.022	270	0.050	270	0.045	272	0.231 ***	272	0.142 *	267	0.167 **
Carotenes/TAG *	259	−0.146	259	−0.050	266	−0.085	263	−0.170 **	263	−0.171 **	266	0.006	266	NA	261	0.285 ***
Tocopherols/TAG *	265	0.015	265	0.014	272	−0.094	269	−0.237 ***	269	−0.239 ***	272	0.039	272	NA	267	0.389 ***
TAC (mM eq.Trolox)	376	−0.063	376	−0.061	385	−0.023	377	0.009	376	0.002	386	0.026	386	0.162 **	382	−0.118 *
ox-LDL (mg/L) *	179	0.009	179	−0.042	184	0.187 *	182	0.094	182	0.118	184	−0.068	184	0.065	183	−0.038
**Total sample**																
Retinol (μg/dL)	936	0.036	936	0.012	978	0.071 *	948	0.057	948	0.055	977	0.217 ***	977	0.142 ***	962	−0.006
Carotenes/TAG *	929	−0.059	929	−0.050	971	−0.075 *	941	−0.189 ***	941	−0.185 ***	971	0.031	971	NA	956	0.285 ***
Tocopherols/TAG *	935	−0.041	935	−0.053	977	−0.113 ***	947	−0.236 ***	947	−0.233 ***	977	0.026	977	NA	962	0.357 ***
TAC (mM eq.Trolox)	1116	−0.087 **	1116	−0.052	1165	0.014	1122	0.019	1120	0.020	1167	0.025	1167	0.087 **	1161	−0.086 **
ox-LDL (mg/L) *	728	0.043	728	0.040	766	0.073 *	742	0.018	742	0.025	766	−0.002	766	0.059	763	−0.104 **

Partial correlation adjusted for sex, age and body mass index in the total samples and separately by pubertal stage, asterisks indicate statistical significance (* *p* < 0.05, ** *p* < 0.01, *** *p* < 0.001). * Ln-transformed variable used for analyses. DBP: diastolic blood pressure; Chol: cholesterol; HDL-C: high-density lipoprotein cholesterol; HOMA-IR: homeostasis model assessment for insulin resistance; NA: non-applicable; Ox-LDL: oxidized low-density lipoprotein; TAC: total antioxidant capacity; TAG: triacylglycerols; SBP: systolic blood pressure.

**Table 5:** Association of plasma antioxidants and oxidative stress biomarkers with pro-inflammatory and endothelial damage biomarkers.

	hsCRP (mg/L)		HGF (µg/L)		IL-6 (ng/L)		IL-8 (ng/L)		MCP-1 (ng/L)		TNFα (ng/L)		aPAI-1 (µg/L)		tPAI-1 (µg/L)		MMP-9 (µg/L)		MPO (µg/L)	
	Beta	*p*	Beta	*p*	Beta	*p*	Beta	*p*	Beta	*p*	Beta	*p*	Beta	*p*	Beta	*p*	Beta	*p*	Beta	*p*
**Prepubertal Children**																				
Retinol (μg/dL)	−0.149	1.2 × 10^−5^	0.087	0.049	-	-	-	-	0.233	4.2 × 10^−8^	0.121	0.004	0.162	2.9 × 10^−5^	0.103	0.011	-	-	−0.166	7.3 × 10^−5^
Carotenes/TAG	-	-	-	-	-	-	-	-	-	-	-	-	−0.096	0.044	−0.190	1.5 × 10^−5^	-	-	-	-
Tocopherols/TAG	-	-	-	-	-	-	-	-	-	-	0.140	0.001	−0.087	0.059	-	-	−0.137	0.002	-	-
TAC (mM eq.Trolox)	-	-	-	-	-	-	-	-	-	-	-	-	-	-	-	-	-	-	−0.143	7.2 × 10^−4^
ox-LDL (mg/L)	-	-	-	-	-	-	-	-	-	-	-	-	0.097	0.010	0.265	6.5 × 10^−11^	-	-	-	-
**Pubertal children**																				
Retinol (μg/dL)	-	-	-	-	-	-	-	-	-	-	-	-	-	-	-	-	-	-	-	-
Carotenes/TAG	-	-	-	-	-	-	-	-	-	-	-	-	0.093	0.003	-	-	-	-	-	-
Tocopherols/TAG	-	-	-	-	-	-	−0.319	5.2 × 10^−5^	-	-	-	-	-	-	-	-	0.166	0.035	-	-
TAC (mM eq.Trolox)	-	-	-	-	0.181	0.020	-	-	0.367	8.6 × 10^−7^	0.281	6.9 × 10^−5^	-	-	−0.218	0.002	-	-	-	-
ox-LDL (mg/L)	-	-	0.162	0.036	-	-	-	-	0.163	0.022	-	-	-	-	0.350	7.7 × 10^−7^	-	-	-	-
**Total sample**																				
Retinol (μg/dL)	−0.124	1.1 × 10^−4^	-	-	-	-	-	-	0.200	1.4 × 10^−7^	0.075	0.045	0.094	0.007	-	-	-	-	−0.102	0.008
Carotenes/TAG	-	-	-	-	-	-	-	-	-	-	−0.093	0.036	−0.162	9.2 × 10^−6^	−0.162	1.7 × 10^−5^	-	-	-	-
Tocopherols/TAG	-	-	-	-	-	-	−0.285	0.043	-	-	0.121	0.005	-	-	-	-	-	-	-	-
TAC (mM eq.Trolox)	-	-	-	-	-	-	-	-	0.158	7.9 × 10^−6^	0.095	0.006	-	-	−0.098	0.004	-	-	−0.116	0.002
ox-LDL (mg/L)	-	-	-	-	-	-	-	-	0.077	0.031	−0.069	0.050	0.094	0.005	0.265	3.1 × 10^−14^	0.100	0.006	-	-

Stepwise multiple regression analyses were performed for each pro-inflammatory/endothelial damage biomarker (dependent variables) using sex, age, body mass index, center and the five antioxidant/oxidative stress markers (independent variables), in the total samples and separately by pubertal stage. Standardized beta (beta) and significance value (*p*) are indicated for those variables that remained in the models. All variables were logarithmically transformed for analyses except retinol, TAC and MPO. HGF: hepatocyte growth factor; hsCRP: high sensitivity CRP; IL-6: interleukin 6; IL-8: interleukin 8; MCP-1: monocyte chemoattractant protein 1; MMP-9: matrix metalloproteinase 9; MPO: myeloperoxidase; Ox-LDL: oxidized low-density lipoprotein; TAC: total antioxidant capacity; TAG: triacylglycerols; TNFα: tumor necrosis factor alpha; tPAI-1: total plasminogen activator inhibitor 1.

## Supplementary Materials

The following are available online at https://www.mdpi.com/2076-3921/9/7/618/s1, Table S1: Descriptive characteristics of the studied population classified by sex and pubertal stage, Table S2: Fulfillment of the individual criteria considered in the classification of metabolically unhealthy status in children according to pubertal stage, Table S3: Plasma antioxidant vitamins and ox-LDL concentrations according to weight and pubertal status, Table S4: Partial correlation analyses between plasma antioxidant and oxidative stress biomarkers and plasma pro-inflammatory and endothelial damage biomarkers.

## Appendix A

Descriptive characteristics of the participants according to sex and pubertal stage are shown in Table S1. The observed plasma retinol and tocopherols concentrations fitted in the range of adequate levels according to previous data, reported to be 20–65 µg/dL for retinol and 0.7–1.6 mg/dL for tocopherols (Gil, 2017). However, our results indicate a highly prevalent deficiency of carotenes (96.9% in normal-weight individuals and 100% in participants with overweight or obesity), for which the reference is established to be >40 µg/dL. Prepubertal females were younger and showed lower weight, height, BMI Z-score, HDL-C, fasting plasma glucose concentrations and tocopherols/TAG ratio, and higher serum TAG and insulin concentrations and TAC values than prepubertal males. At pubertal stage, females also showed lower height, SBP and plasma TAC values and retinol, MCP-1 and TNFα concentrations, and higher insulin and HOMA-IR values than males. Differences between prepubertal and pubertal males were found in anthropometric values, SBP, TAG, fasting glucose, insulin, HOMA-IR, retinol, TAC, aPAI-1 and tPAI-1, which were higher, and BMI Z-score, total cholesterol, HDL-C, carotenes, carotenes/TAG, tocopherols/TAG, IL-6, MCP-1, TNFα and MMP-9, which were lower in pubertal than in prepubertal males. The same differences were observed between prepubertal and pubertal females, except for tocopherols/TAG, ox-LDL, hsCRP and IL-8, which were lower in pubertal than prepubertal females and the BMI Z-score, total cholesterol, TAG, TAC, tPAI-1 and MMP-9 values, that were similar between pubertal and prepubertal females (Table S1).
