# Supplementary material for: Antioxidants and Oxidative Stress in Children: Influence of Puberty and Metabolically Unhealthy Status
Source: Antioxidants (Basel). 2020 Jul 15;9(7):618. doi: 10.3390/antiox9070618 (PMC7402162; doi:10.3390/antiox9070618)
Supplement: Supplementary file 1 [file antioxidants-09-00618-s001.pdf]

Table S1. Descriptive characteristics of the studied population classified by sex and pubertal stage.

		Males			Females		
		N	Mean (%)	SD	N	Mean (%)	SD
Age (y)	Prepubertal	511	9.1^{a}	1.9	442	8.4^{a*}	1.8
	Pubertal	195	12.9^{b}	1.6	296	12.6^{b}	1.9
Weight (kg)	Prepubertal	511	43.1^{a}	15.6	442	39.6^{a*}	12.5
	Pubertal	195	64.6^{b}	18.7	296	63.1^{b}	16.9
Height (m)	Prepubertal	511	1.37^{a}	0.12	442	1.32^{a*}	0.11
	Pubertal	195	1.59^{b}	0.10	296	1.55^{b*}	0.09
BMI (kg/m^2)	Prepubertal	511	22.63^{a}	5.64	442	22.21^{a}	5.02
	Pubertal	195	25.30^{b}	5.42	296	25.94^{b}	5.51
BMI Z-score	Prepubertal	511	2.10^{a}	2.26	442	1.69^{a*}	1.71
	Pubertal	195	1.73^{b}	1.54	296	1.88^{a}	1.55
Normal-weight status	Prepubertal	166	32.5%		124	28.1%	
	Pubertal	52	26.7%		64	21.6%	
Overweight status	Prepubertal	88	17.2%		100	22.6%	
	Pubertal	61	31.3%		109	36.8%	
Obesity status	Prepubertal	257	50.3%		218	49.3%	
	Pubertal	82	42.1%		123	41.6%	
MU status	Prepubertal	251	52.2%		239	57.6%	
	Pubertal	113	61.1%		164	56.7%	
SBP (mm Hg)	Prepubertal	487	105^{a}	15	422	104^{a}	13
	Pubertal	191	114^{b}	14	287	111^{b*}	13
DBP (mm Hg)	Prepubertal	487	64^{a}	12	422	65^{a}	11
	Pubertal	191	67^{b}	9	287	67^{b}	11
Total cholesterol (mg/dL)	Prepubertal	511	168^{a}	31	441	168^{a}	29
	Pubertal	195	162^{b}	29	294	164^{a}	30
HDL-C (mg/dL)	Prepubertal	507	58^{a}	15	435	54^{a*}	14
	Pubertal	191	50^{b}	12	291	50^{b}	13
TAG (mg/dL)	Prepubertal	511	64^{a}	33	441	70^{a*}	37
	Pubertal	195	71^{b}	37	294	73^{a}	34
Fasting glucose (mg/dL)	Prepubertal	510	85^{a}	7	441	83^{a*}	7
	Pubertal	195	87^{b}	8	294	86^{b}	7
Insulin (mU/L)	Prepubertal	490	8.30^{a}	6.12	418	9.19^{a*}	6.91
	Pubertal	185	12.58^{b}	8.27	295	15.07^{b*}	8.42
HOMA-IR	Prepubertal	489	1.76^{a}	1.40	418	1.92^{a}	1.58
	Pubertal	185	2.75^{b}	1.91	294	3.22^{b*}	1.83
Retinol (µg/dL)	Prepubertal	376	26.6^{a}	6.8	329	25.6^{a}	7.3
	Pubertal	109	32.5^{b}	7.6	170	29.9^{b*}	9.0
Carotenes (µg/dL)	Prepubertal	376	11.1^{a}	8.8	329	11.0^{a}	9.0
	Pubertal	108	8.9^{b}	8.3	165	8.3^{b}	5.4
Carotenes/TAG 10^4	Prepubertal	376	21.28^{a}	19.77	329	19.20^{a}	19.73
	Pubertal	108	15.29^{b}	16.22	164	13.97^{b}	10.43
Tocopherols (mg/dL)	Prepubertal	376	0.815^{a}	0.191	329	0.818^{a}	0.220
	Pubertal	109	0.808^{a}	0.226	170	0.772^{b}	0.239
Tocopherols/TAG 10^3	Prepubertal	376	1.48^{a}	0.67	330	1.37^{a*}	0.61
	Pubertal	109	1.33^{b}	0.62	168	1.26^{b}	0.61
TAC (mM eq. Trolox)	Prepubertal	423	1.88^{a}	0.78	358	2.04^{a*}	0.85
	Pubertal	156	2.28^{b}	0.88	235	2.08^{a*}	0.91
Ox-LDL (mg/L)	Prepubertal	313	1.97^{a}	1.96	270	1.92^{a}	1.79
	Pubertal	72	1.80^{a}	1.72	117	1.33^{b}	1.54
hsCRP (mg/L)	Prepubertal	456	2.42^{a}	4.05	391	2.86^{a}	4.68

		Males			Females		
		N	Mean (%)	SD	N	Mean (%)	SD
	Pubertal	161	1.95[a]	2.53	250	1.99[b]	3.23
HGF (μg/L)	Prepubertal	340	0.63[a]	0.50	287	0.64[a]	0.57
	Pubertal	98	0.59[a]	0.38	141	0.58[a]	0.62
IL-6 (ng/L)	Prepubertal	395	5.25[a]	10.08	348	5.10[a]	10.02
	Pubertal	139	3.36[b]	6.09	192	3.32[b]	5.84
IL-8 (ng/L)	Prepubertal	476	2.04[a]	2.42	415	1.99[a]	1.89
	Pubertal	178	1.76[a]	1.40	267	1.58[b]	1.15
MCP-1 (ng/L)	Prepubertal	487	105.37[a]	50.48	419	106.80[a]	52.94
	Pubertal	185	96.72[b]	43.14	276	84.27[b*]	48.51
TNFα (ng/L)	Prepubertal	486	3.53[a]	2.01	419	3.48[a]	2.02
	Pubertal	186	3.03[b]	1.65	275	2.37[b*]	1.56
aPAI-1 (μg/L)	Prepubertal	350	8.87[a]	10.40	304	9.94[a]	10.04
	Pubertal	100	11.71[b]	13.28	152	14.46[b]	16.63
tPAI-1 (μg/L)	Prepubertal	487	22.27[a]	15.58	416	23.91[a]	16.66
	Pubertal	184	24.91[b]	15.22	271	25.16[a]	16.20
MMP-9 (μg/L)	Prepubertal	352	87.89[a]	60.75	302	84.36[a]	53.18
	Pubertal	96	72.45[b]	40.51	148	74.31[a]	61.39
MPO (μg/L)	Prepubertal	466	-0.15[a]	0.52	406	-0.11[a]	0.46
	Pubertal	178	-0.15[a]	0.46	260	-0.10[a]	0.48

Significant differences in the student's t-test (P<0.05) between prepubertal and pubertal children within the same sex are shown with different superscript letters (a, b), and with an asterisk (*) for differences between males and females within the same pubertal stage. aPAI-1: active plasminogen activator inhibitor 1; BMI: body mass index; DBP: diastolic blood pressure; HDL-C: high-density lipoprotein cholesterol; HGF: hepatocyte growth factor; HOMA-IR: homeostasis model assessment for insulin resistance; hsCRP: high sensitivity CRP; IL-6: interleukin 6; IL-8: interleukin 8; MCP-1: monocyte chemoattractant protein 1; MMP-9: matrix metalloproteinase 9; MPO: myeloperoxidase; MU: metabolically unhealthy; Ox-LDL: oxidized low-density lipoprotein; SBP: systolic blood pressure; SD: standard deviation; TAC: total antioxidant capacity; TAG: triacylglycerols; TNFα: tumor necrosis factor alpha; tPAI-1: total plasminogen activator inhibitor 1.

Table S2. Fulfillment of the individual criteria considered in the classification of metabolically unhealthy status in children according to pubertal stage.

	**Prepubertal**		**Pubertal**	
	**N**	**%**	**N**	**%**
MU status (one or more criteria):	490	100%	277	100%
SBP≥90th percentile for age, sex and height	268	55%	127	46%
DBP≥90th percentile for age, sex and height	193	39%	76	27%
Glucose ≥100 mg/dL	25	5%	20	7%
TAG>90th percentile for age, sex and race	126	26%	60	22%
HDL-C <10th percentile for age and sex	72	15%	51	18%
HOMA-IR ≥2.5 (prepubertal) or HOMA-IR ≥3.38 ♂ / ≥3.905 ♀ (pubertal)	196	40%	124	45%

DBP: diastolic blood pressure; HDL-C: serum high-density lipoprotein cholesterol; HOMA-IR: homeostatic model assessment for insulin resistance; MU: metabolically unhealthy; SBP: systolic or diastolic; TAG: serum triacylglycerols.

Table S3. Plasma antioxidant vitamins and ox-LDL concentrations according to weight and pubertal status.

	Prepubertal							Pubertal						
	Normal-weight			Overweight/obesity				Normal-weight			Overweight/obesity			
	**N**	**Mean**	**SD**	**N**	**Mean**	**SD**	**P**	**N**	**Mean**	**SD**	**N**	**Mean**	**SD**	**P**
Retinol (µg/dL)	225	25.0	6.5	480	26.7	7.2	0.001	55	31.2	8.4	224	30.8	8.6	0.873
Carotene/TAG 10E4	225	30.31	26.04	480	15.63	13.72	0.000	55	22.98	19.25	217	12.34	9.86	0.000
Tocopherols/TAG 10E3	226	16.5	6.5	480	13.3	6.1	0.000	55	16.3	6.6	222	12.0	5.7	0.000
TAC (mM eq. Trolox)	257	2.10	0.75	524	1.88**	0.840	0.000	102	1.91	0.71	289	2.25**	0.94	0.000
Ox-LDL (mg/L)	205	2.05	2.06	378	1.89	1.78	0.435	42	1.72	1.79	147	1.45	1.57	0.906

Mean and standard deviation (SD) of the studied biomarkers for children with normal-weight or overweigh/obesity, stratified by pubertal status. P value indicate statistical significance of the general linear model adjusted by sex, age, center for weight status (independent variable) and individual biomarkers (dependent variables). **Statistical significance ($P<0.01$) of the general linear model (adjusted for sex, age, recruitment center and BMI) between prepubertal and pubertal children within each weight category. Carotene/TAG, Tocopherols/TAG and ox-LDL were ln-transformed variables used statistical analyses. Ox-LDL: oxidized low-density lipoprotein; TAC: total antioxidant capacity; TAG: triacylglycerols.

Table S4. Partial correlation analyses between plasma antioxidant and oxidative stress biomarkers and plasma pro-inflammatory and endothelial damage biomarkers.

	hsCRP (mg/L)		HGF (μg/L)		IL-6 (ng/L)		IL-8 (ng/L)		MCP-1 (ng/L)		TNFα (ng/L)		aPAI-1 (μg/L)		tPAI-1 (μg/L)		MMP-9 (μg/L)		MPO (μg/L)	
	**N**	**Corr.**	**N**	**Corr.**	**N**	**Corr.**	**N**	**Corr.**	**N**	**Corr.**	**N**	**Corr.**	**N**	**Corr.**	**N**	**Corr.**	**N**	**Corr.**	**N**	**Corr.**
**Prepubertal children**																				
Retinol (μg/dL)	676	-.117***	617	.011	626	.015	639	-.021	653	.161***	652	.085*	644	.147***	650	.043	644	-.034	620	-.166***
Carotenes/TAG	676	-.078*	617	.014	626	-.026	639	.043	653	.004	652	.048	644	-.128**	650	-.145***	644	-.049	620	-.084*
Tocopherols/TAG	676	.023	617	.066	626	.031	639	.002	653	.103**	652	.135***	644	-.111**	650	-.119**	644	-.116**	620	-.091*
TAC (mM eq.Trolox)	676	-.038	549	.062	644	-.035	760	-.034	774	.096**	774	.032	576	.039	772	.023	576	.062	741	-.133***
ox-LDL (mg/L)	559	.057	546	-.031	549	.034	561	-.012	575	.007	575	-.101*	574	.103*	573	.281***	574	.075	542	.084
**Pubertal children**																				
Retinol (μg/dL)	256	-.071	231	-.152*	231	-.007	229	-.191**	245	.058	244	-.024	244	-.001	238	-.112	236	.007	225	.061
Carotenes/TAG	250	-.047	226	-.08	226	.031	224	-.063	240	-.078	239	-.015	239	-.181**	233	-.071	231	.105	221	-.006
Tocopherols/TAG	256	.013	231	-.105	231	-.008	229	-.259***	245	-.083	244	-.024	244	-.112	238	-.076	236	-.053	225	-.030
TAC (mM eq.Trolox)	309	.079	169	.059	257	.054	367	.102	383	.218***	384	.164**	182	.013	378	-.033	175	-.028	364	-.003
ox-LDL (mg/L)	170	.011	168	.204**	168	-.04	165	.04	181	.119	182	-.104	181	.154*	176	.392***	174	.154*	166	.007
**Total sample**																				
Retinol (μg/dL)	937	-.117***	853	-.041	862	.013	873	-.075*	903	.111***	901	.043	893	.096***	893	-.001	885	-.028	850	-.111**
Carotenes/TAG	931	-.078*	848	-.012	857	-.023	868	.014	898	-.017	896	.022	888	-.142***	888	-.124***	880	-.010	846	-.063
Tocopherols/TAG	937	.014	853	.016	862	.019	873	-.077*	903	.041	901	.079*	893	-.120***	893	-.104 **	885	-.099**	850	-.073*
TAC (mM eq.Trolox)	990	-.018	723	.057	906	-.018	1132	.005	1162	.147***	1163	.073*	763	-.003	1155	.007	756	.054	1110	-.091**
ox-LDL (mg/L)	734	.044	719	.021	722	.016	731	-.007	761	.041	762	-.101**	760	.113**	754	.311***	753	.092*	713	.068

Partial correlation adjusted for sex, age and body mass index in the total samples and separately by pubertal stage (*P<0.05, **P<0.01 y ***P<0.001). All variables were logarithmically transformed for analyses except retinol, TAC and MPO. HGF: hepatocyte growth factor; hsCRP: high sensitivity CRP; IL-6: interleukin 6; IL-8: interleukin 8; MCP-1: monocyte chemoattractant protein 1; MMP-9: matrix metalloproteinase 9; MPO: myeloperoxidase; Ox-LDL: oxidized low-density lipoprotein; TAC: total antioxidant capacity; TAG: triacylglycerols; TNFα: tumor necrosis factor alpha; tPAI-1: total plasminogen activator inhibitor 1.
